# Gems of Journal Literature

**Published:** 1896-08

**Authors:** 


Some Gems of Journal Literature.—Men sometimes unintentionally say "good things;" good in a different sense from that intended. For instance, a writer may unintentionally make a pun, or perpetrate a joke, or sometimes, when he attempts the stilted style, he may make a "flummux." There is but a step from the sublime to the ridiculous, and in writing, as in acting, if a thing be overdone, ever so little, it becomes a burlesque. We propose to give a few illustrations, taken from our Texas contemporaries. As "full many a gem of purest ray serene the deep unfathomed caves of ocean bear," so full many an editorial gem, (?) serene or otherwise,—can be exhumed from the unread pages of some ambitious little publication. One of our contemporaries for July, with an *away-out-yonder*-name, is a treasure trove, a veritable "find." It is remarkable that men will write for publication, whose lack of knowledge of the fundamental rules of rhetoric,—nay, of orthography constantly exposes them to criticism,—yet they do; and the funny part of their productions is, that they are unintentionally funny. A critcism (?) in bad English is a joke, within itself; a reminiscence of Artemus Ward.

In an editorial on medical legislation and homeopathy, in which the writer intends to be very severe (?) on the "Red Back," one of the editors of the publication referred to says, amongst other good things:

"We do not believe in the theory of Similiar Similibus Curanter; only a very few people do, and of those few who profess to, most of them simply believe in less drugs."

Lest it might be thought that he meant the old familiar *s. s. curantur*, and only spelled it incorrectly, the writer kindly explains that the theory above cited means less drugs. Good. Thanks, doctor.

Continuing the discussion of homeopathy (*loc. cit.*) our unconsciously waggish contemporary says:

"While we do not believe in the homeopathic theory, we do not believe in denominating them quacks."

Denominating "them,"—whom? quacks.

Another; better still:

"Quackery is not confined to the ignorant, and together with his knowledge [whose knowledge?] and unctious money, he [who?] generally glides through the examining boards."

"Unctious money" *is* good,—especially in connection with the "gliding" process; it doubtless refers to "filthy lucre," and indicates that boards yet unappointed, are open to bribery; an unwarranted arraignment of the entire profession.

"Our objection to the proposed medical law for the State," says our little joker (*loc. cit.*) "is, that you [who?] cannot shut out quackery."

Well, now, we submit that because some unknown of the second person-spoken-to cannot "shut out quackery," that is no valid reason for condemning the law. Our friend is unjust. Our only objection to Jones is that Smith cannot control his temper,—is a parallel.

Honesty Not the Best Policy. — The editorial writer above quoted, continuing the subject of homeopathy and quacks, says, (*loc. cit.*):

"The greater number of mal-treated cases coming under our observation was [were] the fault of honesty and not of igno-ignorance."

Respectfully submitted without comment.

But here are some "gems" of composition (from the same article), which are too good to be lost, or buried in a small circulation, and the "Red Back" gives them to the world that they may be incorporated in the future text books on rhetoric as specimens:

"If every man now practicing medicine in Texas, held a license to practice, by giving the accused the benefit of the doubt, which the law always does, this bill [meaning the State Medical Association's proposed bill for a mixed board], were it a law, would not shut out five of the many quacks practicing medicine in Texas to-day."

That can't be improved upon. What doubt? What accused?

"It has been our observation that the quack is always on the inside of the fence. If he can not get in by his knowledge and gold-salve unction, used on the board as before mentioned, he sometimes grows up inside the pasture."

Now, it does seem that if "he" is "always on the inside of the fence," it would require neither "knowledge" nor "gold-salve unction" [whatever that may be] to help him get in. There is something decidedly Weggish and Boffinsish in that sentence, especially the "before mentioned."

The doctor gets "boards" and "fences" and "pastures" mixed up in a way that indicates a better acquaintance with the farm than with the forum. The above, analyzed, is as if to say: If a man can not get inside of an enclosure, being already in; if he can not climb over the fence nor slip through the "boards," he will work himself in (by algebra, we suppose) and just *grow* up, Topsy-fashion.

Leaving medical legislation, after the above lucid comments, our contemporary turns his attention to archæological matters, and in describing the finding of some skeletons in a shell bank near Galveston, says:

"It is most probably the burying ground of some tribe of Indians long since extinct, and they have left their matchless ivory teeth, bleached bones and buck-horn trinkets to remind us that the sea may some day come quietly, noiselessly 'neath nature's deep base and lay tiny shells on our graves till they are ruthlessly brushed away by some future Vandal's steam shovel. SEE?"

Yes; we see. "Noiselessly 'neath nature's deep base" is poetical, and "laying tiny shells on our graves" is decidedly sentimental, but having the tiny shells *brushed* away by a *steam* shovel is a metaphor that is hard to classify; while we protest that the job-lot of buck-horn and ivory smacks too much of trade, and suggests the bargain counter too much to be found in such company; it takes all the poetry out of it.

